# InfoKids+: A Validation Study of a Pediatric Acuity Risk Stratification Algorithm

**DOI:** 10.1016/j.mcpdig.2025.100220

**Published:** 2025-04-15

**Authors:** Carl A. Starvaggi, Sophie Affentranger, Noelie Lengeler, Johan N. Siebert, Annick Galetto-Lacour, Rainer Tan, Manon Jaboyedoff, Claudia E. Kuehni, Mary-Anne Hartley, Kristina Keitel

**Affiliations:** a Division of Pediatric Emergency Medicine, Department of Pediatrics, Inselspital, Bern University Hospital, Bern, Switzerland; b Division of Pediatric Respiratory Medicine and Allergology, Department of Pediatrics, Inselspital, Bern University Hospital, Bern, Switzerland; c Department of Pediatrics, Inselspital, Bern University Hospital, Bern, Switzerland; d Department of Orthopedic Surgery and Traumatology, Spitalzentrum Oberwallis, Brig, Switzerland; e Graduate School for Health Sciences, University of Bern, Bern, Switzerland; f Institute of Social and Preventive Medicine, University of Bern, Bern, Switzerland; g Department of Pediatrics, Children’s Hospital Lucerne, Lucerne Cantonal Hospital, Lucerne, Switzerland; h Department of Pediatric Emergency Medicine, Geneva Children’s Hospital, Geneva, University Hospitals, Geneva, Switzerland; i Unisanté, Center for Primary Care and Public Health, University of Lausanne, Lausanne, Switzerland; j Swiss Tropical and Public Health Institute, Allschwil, Switzerland; k Pediatric Infectious Diseases and Vaccinology Unit, Service of Pediatrics, Department Women-Mother-Child, Lausanne University Hospital and University of Lausanne, Lausanne, Switzerland; l Laboratory for intelligent Global Health and Humanitarian Response Technologies (LiGHT), Yale School of Medicine, Department of Biomedical Informatics and Data Science, New Haven, CT; m Laboratory for intelligent Global Health and Humanitarian Response Technologies (LiGHT), EPFL, School of Computer Science, Lausanne, Switzerland

## Abstract

**Objective:**

To prospectively validate InfoKids+, a pediatric acuity electronic risk stratification algorithm (eRSA), against a nurse-based triage standard (nbTS).

**Participants and Methods:**

We conducted a prospective validation study in a Swiss university hospital pediatric emergency department to assess the performance of a pediatric acuity eRSA, InfoKids+, on the basis of a well-established parental guidance application, InfoKids. Participants completed the eRSA once seated in a consultation booth. We compared the acuity levels from InfoKids+ (urgent, <4 hours; nonurgent, <24 hours; and no emergency, ≥24 hours) against an nbTS. The primary outcome was the level of agreement and rate of alignment between InfoKids+ and the reference standard.

**Results:**

We included 1990 participants from June 3, 2020, through January 31, 2022. InfoKids+ showed a slight level of agreement with the nbTS (κ_lw_=0.08; 95% CI, 0.06-0.10). InfoKids+ triaged 1762 (89%) cases as urgent (<4 hours), 106 (5%) as nonurgent (≤24 hours), and 122 (6%) as no emergency (≥24 hours), compared with 810 (41%), 843 (42%), and 337 (17%) triages by the nbTS, respectively (*P*<.001). InfoKids+ acuity level aligned with the reference standard in 888 (45%) cases, whereas it overreferred and underreferred in 999 (50%) and 103 (5%) cases, respectively (*P*<.001).

**Conclusion:**

In summary, our study uncovered notable discrepancies between the InfoKids+ algorithmic triage and conventional nurse-based triage. Our results highlight the critical need for rigorous validation of such tools for accuracy and safety before public release to ensure these tools are beneficial and do not inadvertently cause harm or misallocation of resources.

In recent years, pediatric emergency departments (PEDs) have witnessed a marked increase in visits, with low-acuity cases largely contributing to this rise.^1^ In 2018, low-acuity cases accounted for 54% of all PED visits in 2 Swiss university hospitals.^2^ This trend coincides with the rising use of online health resources, including web-based and smartphone-driven self-diagnosis tools.^3^ These tools have raised concerns due to their lack of validation and opaque underlying logic.^4^ Notably, a review encompassing 15 primary care self-triage instruments revealed inaccurate triage advice for 43% of clinical scenarios, underscoring the need for more reliable tools.^4^

Besides self-diagnosis tools, electronic risk stratification algorithms (eRSAs) have emerged as user-friendly interfaces that not only provide probabilistic guidance but also facilitate continuous data collection to enable monitoring for effectiveness improvements. In light of a 2020 directive from the European Union, validation of the safety and efficacy of eRSA guidance should be performed before routine implementation.^5^^,^^6^ Robust validation processes are increasingly important in the era of artificial intelligence where the logic of algorithms is less interpretable and for which continuous learning needs to be anchored to meaningful clinical outcomes.^7^

Our study aimed to focus on InfoKids, a digital information mobile application developed by the Geneva University Hospitals PED team for caregivers, addressing common pediatric urgent care concerns.^8^ Since its publication in 2015, the application has been downloaded over 70,000 times, showing good to excellent usability.^9^ However, the guidance of InfoKids has not been previously evaluated. We therefore transformed the content of the InfoKids application into an eRSA, InfoKids+, and compared its acuity level assignment with routine triage.^10^

The primary goal of our study was to prospectively validate InfoKids+ to accurately determine the acuity level in pediatric emergency situations. Secondary objectives were (1) evaluating factors influencing the interpretation and future adoption of InfoKids+ by caregivers and (2) collecting data for the development of an evidence-based enhancement of InfoKids+, preparing it for potential implementation in routine practice.

## Participants and Methods

We conducted a prospective validation study at the Bern University Hospital PED, located in the German-speaking part of Switzerland. Serving a catchment area of approximately 400,000 children, the PED handles around 27,000 visits annually.^11^ The study’s primary goal was to assess the performance of InfoKids+, an eRSA, in determining the urgency level of pediatric patients’ conditions. We compared this assessment against a nurse-based triage standard (nbTS). We also explored symptom groups and sociodemographic characteristics associated with incorrect acuity level assignments. The data collection period spanned 20 months, from June 3, 2020 through January 31, 2022, coinciding with the COVID-19 pandemic in Switzerland.

### Participants

Participants included all legal guardians of children aged 0 to 16 years visiting the PED. We obtained written informed consent and additional assent from children aged older than 13 years. The study did not use specific exclusion criteria. Participant recruitment was continuous, with the research team available onsite during varied hours throughout the 24-hour day. This study was approved by the Bernese Cantonal Research Ethics Committee.

We aimed at enrolling 2000 patients with an estimated distribution of the acuity level on the basis of routine data from the PED: 40% urgent (<4 hours), 50% nonurgent (<24 hours), and 10% no emergency (≥24 hours). Using normograms calculations proposed by Buderer,^12^ we aimed for the following sample sizes for each acuity level (α=0.05, absolute precision=0.07): 800 urgent (<4 hours), 150 nonurgent (<24 hours), and 900 no emergency (≥24 hours), resulting in 1850 participants.

### InfoKids+ eRSA (Index Test)

The InfoKids+ eRSA served as the index test for assessing the acuity level. We developed an electronic case report form (eCRF), on the basis of the well-established InfoKids application, to mirror InfoKids+ question structure (Supplemental Figure 1, available online at https://www.mcpdigitalhealth.org).^8^^,^^10^ We incorporated chief complaints, symptom groups, questions, and acuity levels from the original application into the eCRF.^10^ It is important to note that the acuity levels given by the InfoKids application are not on the basis of the Australasian Triage Scale (ATS) used in Bern nor the Canadian Triage and Acuity Scale used in Geneva. Participants completed the eCRF once situated within a consultation booth after triage and were instructed to simulate responses as if they still were at home. Using the eCRF entries, the eRSA calculated the acuity level, which remained concealed from patients, parents, and clinical staff. Our study team defined the following 2 acuity levels: (1) urgent (<4 hours), (2) nonurgent (<24 hours), and (3) no emergency (≥24 hours).

### Nurse-Based Triage Standard (Reference Standard)

The reference standard relied on the ATS-based acuity level determined by triage nurses up patients’ arrival at the PED.^13^ To align with InfoKids+’ focus on advising care urgency rather than providing a diagnosis, we used acuity levels from the ATS instead of diagnostic classifications. We defined the same 3 acuity levels for the reference standard as for the index test as described further.

**Figure 1:**
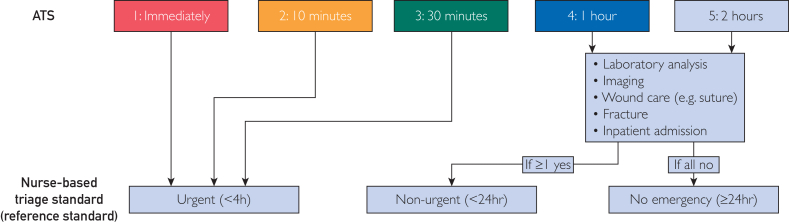
Interconnection between the Australasian Triage Scale (ATS) and the nurse-based triage standard (nbTS) used as reference standard in this study.

The ATS establishes 5 acuity levels: (1) immediate doctor attention, (2) ≤10 minutes, (3) ≤30 minutes, (4) ≤60 minutes, and (5) ≤120 minutes.^13^ Trained triage nurses ascribe these levels to patients upon arrival at the PED. Notably, the ATS lacks a classification for care needed within 24 hours or for self-care. Recognizing that pediatric primary care providers might lack resources to perform certain diagnostic (eg, x-rays) and treatment (eg, sutures) procedures for some nonurgent cases, we defined the reference standard considering the following 3 constraints (Figure 1):

1. Urgent (<4 hours): ATS 1, 2, or 3;
2. Nonurgent (<24 hours): ATS 4 or 5 with laboratory analysis and/or image study and/or wound care and/or fracture and/or inpatient admission;
3. No emergency (≥24 hours): ATS 4 or 5 without any conditions listed in the second point.

### Comparison

We compared the acuity levels coming from the nbTS with the ones generated by InfoKids+. This comparison yielded the following 3 potential outcomes:

• Aligned: InfoKids+ and the nbTS assigned the same acuity level;
• Overreferred: InfoKids+ assigned a higher acuity level than the nbTS;
• Underreferred: InfoKids+ assigned a lower acuity level than the nbTS.

Our study team had access to the ATS level through the electronic patient data system. The eRSA details, however, were exclusively accessible to the study team responsible for data analysis. Time intervals and clinical interventions between the index test and reference standard were not evaluated due to the preclinical nature of InfoKids+.

### Statistical Analyses

We used StataMP16 (StataCorp) to conduct our data analysis. We excluded missing values from our calculations. Descriptive statistics encompassed frequency, percentages, means, standard deviations, and medians. *P*<.05 was considered statistically significant. We compared the acuity levels of InfoKids+ and nbTS using a 3 × 3 table and the Pearson χ^2^ test. To analyze the level of agreement between the index test and the reference standard, we used the linear-weighted κ. We predefined subgroup analyses to explore alignment variations across symptom groups and sociodemographic characteristics using the Pearson χ^2^ test. We did not perform formal adjustments for multiple testing nor any sensitivity analysis. An exploratory subgroup analysis assessed changes in the level of agreement by altering the acuity level definition of InfoKids+ (Supplemental Table 1, available online at https://www.mcpdigitalhealth.org).

## Results

### Participants

**Figure 2:**
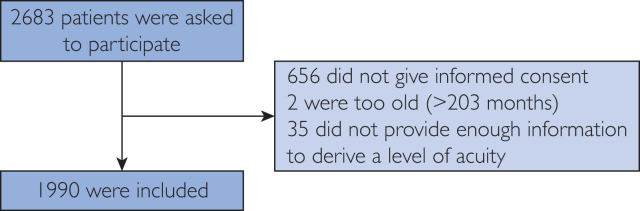
Participant progression. The primary reasons for declining informed consent were as follows (in descending order): personal preference against participation; language barrier; caregiver fatigue; excessive time commitment; inability to provide informed consent; and previous participation (ie, multiple pediatric emergency department visits).

In total, 2683 caregivers were asked to participate whereof 656 (25%) did not give informed consent. However, the core demographic characteristics of this group did not significantly differ from those included (Supplemental Table 2, available online at https://www.mcpdigitalhealth.org). Two (<0.1%) were excluded as their children were older than 16 years, and 35 (<0.1%) because they did not provide enough information to derive an acuity level by InfoKids+. In total, we included 1990 patients from June 3, 2020 through January 31, 2022 (Figure 2).

The median patient age was 70 (IQR, 23-130) months, 18 months older compared with the general median age at the PED during the study period (52 [IQR, 21-107] months; *P*<.001) (Supplemental Table 2). Chronic illness was reported in 9%, and 83% reported they adhered to the Swiss vaccination schedule; 93% of children were born in Switzerland, as were 66% of mothers and fathers, respectively. Further, 49% of parents had a university or higher technical college degree (Table 1).

**Table 1:** Basic Demographic Characteristics of the Study Population

Core demographic characteristic	Values	NA, n (%)
Children (N=1990)		
Age (mo)		
Mean (95% CI)	79.48 (76.94-82.02)	
SD (min, max)	57.81 (0, 199)	
Median (IQR)	70 (25-130)	
Age categories		
<1 mo	9 (0.5)	
1-11 mo	208 (10.5)	
12-23 mo	236 (11.9)	
2-5 y	550 (27.6)	
6-11 y	598 (30.1)	
12-16 y	389 (19.5)	
Sex		
Female	923 (46.4)	
Male	1067 (53.6)	
Birth country		
Switzerland	1846 (92.8)	
Abroad	134 (6.7)	
European	55 (2.8)	
Non-European	79 (4.0)	
Unknown	10 (0.5)	
Chronic illness present (n=1983)^a^		7 (0.4)
No	1803 (90.9)	
Yes	180 (9.1)	
Allergy present (n=1982)^a^		8 (0.4)
No	1733 (87.4)	
Yes	249 (12.6)	
Food allergy	64 (25.7)	
Medication allergy	32 (12.9)	
Other	160 (64.3)	
Vaccination status (n=1981)^a^		9 (0.5)
Fully vaccinated as per the Swiss vaccination plan	1641 (82.8)	
Partially vaccinated	196 (9.9)	
HiB	56 (28.6)	
PCV	58 (29.6)	
Measles	93 (47.5)	
Tetanus	123 (62.8)	
Not at all vaccinated	112 (5.7)	
I do not know	32 (1.6)	
Time of presentation		
Mean (95% CI)	13:47 (13:38-13:56)	
SD (min, max)	03:16 (00:22, 23:29)	
Median (IQR)	13:43 (11:32-15:58)	
06:00-17:59	1764 (88.6)	
18:00-05:59	226 (11.4)	
Caregivers (N=1990)		
Who filled in the eCRF		
Patient	15 (0.8)	
Parents	1846 (92.8)	
Mother	1321 (66.4)	
Father	525 (26.4)	
Family	98 (4.9)	
Mother-in-law or father-in-law	6 (0.3)	
Grandparent	16 (0.8)	
Aunt or uncle	5 (0.3)	
Sibling	71 (3.6)	
Other	22 (1.1)	
Nanny or babysitter	3 (0.2)	
Friend	4 (0.2)	
Other	15 (0.8)	
Unknown	9 (0.5)	
Birth country of mother (n=1528)^a^		462 (23.2)
Switzerland	1016 (66.5)	
Abroad	484 (31.7)	
I do not know or do not want to answer	28 (1.8)	
Birth country of father (n=1513)^a^		477 (24.0)
Switzerland	999 (66.0)	
Abroad	478 (31.6)	
I do not know or do not want to answer	36 (2.4)	
Education of the mother (n=1514)^a^		476 (23.9)
Did not go to school	9 (0.6)	
Mandatory education	120 (7.9)	
Apprenticeship	584 (38.6)	
Higher technical or commercial college	94 (6.2)	
University	614 (40.6)	
I do not know or do not want to answer	93 (6.1)	
Education of the father (n=722)^a^		1268 (63.7)
Did not go to school	5 (0.7)	
Mandatory education	40 (5.5)	
Apprenticeship	243 (33.7)	
Higher technical or commercial college	72 (10.0)	
University	311 (43.1)	
I do not know or do not want to answer	51 (7.1)	
Difficulty paying bills (n=1305)^a^		685 (34.4)
No	1119 (85.8)	
Yes	97 (7.4)	
I do not know or do not want to answer	89 (6.8)	
Financial situation compared with others (n=1299)^a^		691 (34.7)
Above average	322 (24.8)	
Average	706 (54.4)	
Below average	103 (7.9)	
I do not know or do not want to answer	168 (12.9)	

Values are n (%) unless specified.

eCRF, electronic case report form; NA, not applicable.

a Voluntary question.

Approximately 86% expressed no difficulty paying their household bills, and 79% perceived their financial status as average or above compared with other Swiss families. In total, 1764 visits (89%) occurred between 6 am and 6 pm (Table 1); 41% of children in our study were assigned an ATS score of ≤3, which was significantly more compared with 30% of all PED cases during the study period (*P*<.001) (Supplemental Table 2).

### Agreement and Alignment

Table 2 shows the level of agreement and alignment between InfoKids+ and the nbTS. We observed a slight level of agreement (κ_lw_=0.08; 95% CI, 0.06-0.10). InfoKids+ generally assigned higher acuity levels: 1762 (89%) as urgent (<4 hours), 106 (5%) as nonurgent (≤24 hours), and 122 (6%) as no emergency (≥24 hours), compared with 810 (41%), 843 (42%), and 337 (17%) by the nbTS, respectively (*P*<.001). Notably, InfoKids+ missed 29 (1%) urgent (<4 hours) cases, triaging them as no emergency (≥24 hours) (Table 2). InfoKids+’ acuity level aligned with the reference standard in 888 (45%) cases, whereas it overreferred and underreferred 999 (50%) and 103 (5%) cases, respectively (*P*<.001) (Table 2).

**Table 2:** Agreement and Alignment

Reference standard	Urgent (<4 h)	Nonurgent (<24 h)	No emergency (≥24 h)	Total
3 × 3 crosstabulation of agreement				
Index test				
Urgent (<4 h)	771 (38.7)	691 (34.7)	300 (15.1)	1762 (88.5)
Nonurgent (<24 h)	10 (0.5)	88 (4.4)	8 (0.4)	106 (5.3)
No emergency (≥24 h)	29 (1.4)	64 (3.2)	29 (1.5)	122 (6.1)
Total	810 (40.7)	843 (42.4)	337 (16.9)	1990 (100.0)
χ^2^	95.398			
*P*	<.001			
κ_lw_^a^ (95%CI)	0.080 (0.057-0.102)			
3 × 3 crosstabulation of alignment				
Alignment of index test				
Aligned	771 (38.7)	88 (4.4)	29 (1.5)	888 (44.6)
Overreferred	0 (0.0)	691 (34.7)	308 (15.5)	999 (50.2)
Underreferred	39 (2.0)	64 (3.2)	0 (0.0)	103 (5.2)
Total	810 (40.7)	843 (42.4)	337 (16.9)	1990 (100.0)
χ^2^	1.50 × 10^3^			
*P*	<.001			

Values are n (%).

a Linear-weighted κ with the following levels of agreement: <0, no agreement; 0.00-0.20, slight agreement; 0.21-0.40, fair agreement; 0.41-0.60, moderate agreement; 0.61-0.80, substantial agreement; >0.81, almost perfect agreement.

### Subgroup Analysis

Table 3 summarizes the alignment rates of the chief complaint, symptom groups, and sociodemographic characteristics between InfoKids+ and the nbTS; 1108 (56%) participants prompted a medical chief complaint being the reason for their visit (mainly gastrointestinal symptoms and fever). A surgical chief complaint, primarily fractures or injuries, accounted for 882 (44%) visits.

**Table 3:** Alignment by Chief Complaint, Symptom Group, and Sociodemographic Characteristics

Characteristic	Total (N=1990), n (%)	Missing, n (%)	Aligned (n=888), n (%)	Overreferred (n=999), n (%)	Underreferred (n=103), n (%)	χ^2^	*P*	Overall alignment per group compared with all
Chief complaints								
Medical chief complaint^a^	1108 (55.7)	NA	549 (49.5)	530 (47.8)	29 (2.6)	48.000	<.001	**↑**
Musculoskeletal symptoms	60 (3.0)	NA	13 (21.7)	41 (68.3)	6 (10.0)	14.140	.001	**↓**
Diabetes (pees and drinks a lot)	13 (0.7)	NA	6 (46.2)	7 (53.8)	0 (0.0)	0.719	.70	—
ORL symptoms (including ophthalmologic symptoms)	156 (7.8)	NA	63 (40.4)	93 (59.6)	0 (0.0)	12.431	.002	**←→**
Gastrointestinal symptoms	374 (18.8)	NA	167 (44.7)	205 (54.8)	2 (0.5)	21.118	<.001	**←→**
Psychiatric symptoms	16 (0.8)	NA	12 (75.0)	4 (25.0)	0 (0.0)	6.211	.05	**↑**
Fever	359 (18.0)	NA	176 (49.0)	178 (49.6)	5 (1.4)	14.047	.001	**←→**
Headache	131 (6.6)	NA	59 (45.0)	72 (55.0)	0 (0.0)	7.897	.02	**←→**
Malaise or convulsion (including loss of consciousness)	126 (6.3)	NA	92 (73.0)	34 (27.0)	0 (0.0)	45.708	<.001	**↑**
Dermatologic symptoms	80 (4.0)	NA	38 (47.5)	40 (50.0)	2 (2.5)	1.308	.52	—
Cough or difficulty breathing	225 (11.3)	NA	142 (63.1)	83 (36.9)	0 (0.0)	41.517	<.001	**↑**
Urogenital symptoms	70 (3.5)	NA	27 (38.6)	34 (48.6)	9 (12.9)	8.904	.01	**←→**
Something else	155 (7.8)	NA	85 (54.8)	61 (39.4)	9 (5.8)	7.999	.02	**↑**
COVID test	27 (1.4)	NA	11 (40.7)	16 (59.3)	0 (0.0)	1.957	.38	—
Surgical complaint^a^	882 (44.3)	NA	339 (38.4)	469 (53.2)	74 (8.4)	48.000	<.001	**↓**
Ingestion	31 (1.6)	NA	25 (80.6)	6 (19.4)	0 (0.0)	16.756	<.001	**↑**
Burn	13 (0.7)	NA	3 (23.1)	10 (76.9)	0 (0.0)	3.900	.14	—
Cut injury	63 (3.2)	NA	14 (22.2)	46 (73.0)	3 (4.8)	14.084	.001	**↓**
Injured tooth	20 (1.0)	NA	8 (40.0)	11 (55.0)	1 (5.0)	0.191	.91	—
Electric shock	2 (0.1)	NA	1 (50.0)	1 (50.0)	0 (0.0)	0.117	.94	—
Bite wounds	10 (0.5)	NA	2 (20.0)	8 (80.0)	0 (0.0)	3.664	.16	—
Sting	7 (0.4)	NA	1 (14.3)	6 (85.7)	0 (0.0)	3.577	.17	—
Fracture or injury	625 (31.4)	NA	236 (37.8)	342 (54.7)	47 (7.5)	22.998	<.001	**↓**
Something else	178 (8.9)	NA	74 (41.6)	77 (43.3)	27 (15.2)	39.998	<.001	**←→**
Demographic characteristic								
Age under 5 y	1003 (50.4)	NA	480 (47.9)	483 (48.2)	40 (4.0)	11.936	.003	**↑**
Female	938 (47.1)	NA	435 (46.4)	462 (49.3)	41 (4.4)	3.759	.15	—
Child born abroad	134 (6.8)	10 (0.5)	49 (36.6)	80 (59.7)	5 (3.7)	5.237	.07	—
Mother born abroad	484 (32.3)	490 (24.6)	185 (38.2)	281 (58.1)	18 (3.7)	15.574	<.001	**↓**
Father born abroad	478 (32.4)	513 (25.8)	179 (37.4)	276 (57.7)	23 (4.8)	13.708	.001	**↓**
One parent born abroad	645 (43.5)	506 (25.4)	247 (38.3)	371 (57.5)	27 (4.2)	19.147	<.001	**↓**
Both parents born abroad	317 (21.2)	497 (25.0)	117 (36.9)	186 (58.7)	14 (4.4)	10.604	.005	**↓**
Chronic disease present	180 (9.1)	7 (0.4)	111 (61.7)	56 (31.1)	13 (7.2)	28.785	<.001	**↑**
Allergy present	249 (12.6)	8 (0.4)	127 (51.0)	107 (43.0)	15 (6.0)	5.958	.05	—
Fully vaccinated	1641 (84.2)	41 (2.1)	713 (43.4)	841 (51.2)	87 (5.3)	4.989	.08	—
Visit during office hours	1764 (88.6)	NA	780 (44.2)	889 (50.4)	95 (5.4)	2.009	.37	—
Parent filled in the eCRF	1846 (93.2)	9 (0.5)	826 (44.7)	923 (50.0)	97 (5.3)	0.875	.65	—
Mother with only mandatory education or no school	129 (9.1)	569 (28.6)	47 (36.4)	78 (60.5)	4 (3.1)	5.648	.06	—
Father with only mandatory education or no school	45 (6.7)	1319 (66.3)	18 (40.0)	26 (57.8)	1 (2.2)	1.865	.39	—
One parent with only mandatory education or no school	151 (20.0)	1236 (62.1)	57 (37.7)	89 (58.9)	5 (3.3)	4.943	.08	—
Both parents with only mandatory education or no school	23 (1.7)	652 (32.8)	8 (34.8)	15 (65.2)	0 (0.0)	2.805	.25	—
Mother with university degree	614 (43.2)	569 (28.6)	261 (42.5)	317 (51.6)	36 (5.9)	1.050	.59	—
Father with university degree	311 (46.3)	1319 (66.3)	127 (40.8)	161 (51.8)	23 (7.4)	1.492	.47	—
One parent with university degree	711 (73.8)	1027 (51.6)	304 (42.8)	367 (51.6)	40 (5.6)	0.085	.96	—
Both parents with university degree	214 (19.0)	861 (43.3)	84 (39.3)	111 (51.9)	19 (8.9)	5.613	.06	—
Difficulty paying household bills	97 (8.0)	774 (38.9)	43 (44.3)	53 (54.6)	2 (2.1)	2.596	.27	—
Financial situation below average	103 (9.1)	859 (43.2)	39 (37.9)	60 (58.3)	4 (3.9)	2.583	.28	—

Only if *P* (χ2) was statistically significant: • ↑: The alignment was statistically significant better for this group compared with all cases. • ↓: The alignment was statistically significant worse for this group compared with all cases. • **←→**: There is no statistical significance concerning general alignment, but concerning the rate of overreferrals or underreferrals.

eCRF, electronic case report form; NA, not applicable.

a Although the question between medical and surgical chief complaint was a single-choice question, the individual symptom groups per chief complaint (medical or surgical) were part of a multiple choice question.

Within the chief complaint, participants could choose multiple symptom groups. In average, participants chose 1.6 symptom groups per medical and 1.1 symptom groups per surgical chief complaint visit. Further, 333 (17%) participants chose the symptom group “something else”: 155 (14%) within the medical and 178 (20%) within the surgical chief complaint (Table 3). However, 152 (98%) other symptoms within the medical and 168 (94%) within the surgical chief complaint could be attributed to one of the existing symptom groups (Supplemental Table 3, available online at https://www.mcpdigitalhealth.org).

### Chief Complaint and Symptom Groups

The medical chief complaint showed a higher alignment rate (50%; *P*<.001) than the surgical chief complaint (38%, *P*<.001). Notable symptom groups within the medical chief complaint with high alignment rates were psychiatric symptoms (75%; *P*=.01), malaise or convulsion (73%; *P*<.001), and cough or difficulty breathing (63%; *P*<.001). Musculoskeletal symptoms were the only medical symptom group associated with a significantly worse alignment rate (22%; *P*=.001) (Table 3).

Surgical symptom groups with low alignment rates included cut injuries (22%; *P*=.001) and fracture or injury (38%; *P*<.001). Ingestion was the only surgical symptom group associated with a significantly better alignment rate (81%; *P*<.001) (Table 3).

Symptom groups with high alignment rates had significantly higher rates of urgent (<4 hours) triages by the nbTS compared with all cases: 75% (*P*=.005) for psychiatric symptoms, 72% (*P*<.001) for malaise or convulsion, 63% (*P*<.001) for cough or difficulty breathing, and 81% (*P*<.001) for ingestion. In contrast, symptom groups with low alignment rates had significantly lower rates of urgent (<4 hours) nbTS triages: 25% (*P*=.01) for musculoskeletal symptoms, 19% (*P*<.001) for cut injuries, and 25% (*P*<.001) for fracture or injury (Supplemental Table 4, available online at https://www.mcpdigitalhealth.org).

### Sociodemographic Characteristics

Sociodemographic characteristics associated with significantly higher alignment rates were age <5 years (48%; *P*=.003) and having chronic diseases (62%; *P*<.001), whereas foreign-born parents (38% [*P*<.001] for 1 and 37% [*P*=.005] for both parents born abroad) were associated with lower alignment rates (Table 3).

### Safety and Data Usage

We observed no adverse events from the index test or the reference standard. The PED team did not use the index test results in the diagnostic process, remaining accessible solely to the study team.

## Discussion

### Main Findings

Our study assessed the performance of InfoKids+, a pediatric eRSA, in determining acuity levels against nbTS. Our findings highlight the need for rigorous validation of such tools because InfoKids+ exhibited a poor performance, aligning in only 45% of cases with the reference standard. Compared with the reference standard, InfoKids+ exhibited a higher rate of triaging cases as urgent (<4 hours; 41% vs 89%), contributing to a 50% overreferral rate. Underreferrals by InfoKids+ were infrequent, yet they did occur. Although a low rate of underreferrals is typically desired in an online triage tool, it remains uncertain whether such a tool would effectively reduce, or conversely exacerbate, the number of unnecessary PED visits. As shown in another study, tools like InfoKids+ primarily reassure parents that seeking professional care is unnecessary, raising concerns that they may also unintentionally encourage unnecessary PED visits.^14^

InfoKids+ tended to overrefer cases, which was likely primarily due to its conservative algorithm design: clinical findings often found with common PED symptoms (eg, pain and bad general appearance) automatically led to an urgent (<4 hours) triage by InfoKids+ (Supplemental Figure 1). This, however, was intended when designing the eRSA to avoid missing any critical patients and to maximize patient safety.^10^ Altering the acuity level definition of InfoKids+ regarding these clinical signs led to a shift of cases between acuity levels, however not influencing the level of agreement (Supplemental Table 1). Notably, InfoKids+ did not collect any vital signs except for the self-measured temperature, therefore lacking objective parameters to help assess the acuity level. Additionally, the more holistic assessment by a triage nurse, which takes into account various dimensions of a child’s condition beyond just symptom constellations, also contributed to this phenomenon.

Although instances of undertriage were rare, they still pose a notable safety concern for tools like InfoKids+, necessitating vigilant monitoring and rectification before their routine deployment.^9^ A detailed analysis found that the critical underreferrals in this study could mainly be attributed to eRSA logic errors, participants providing inaccurate information, or comprehension errors by participants.^15^ These critical aspects need to be thoroughly addressed before considering the routine implementation of such tools.

Across the different symptom groups, only a few reported markedly better alignment rates with the nbTS, notably in nontraumatic symptom groups. This corresponds to the findings from a similar triage tool where the assessment of nontrauma symptoms was more effective compared with surgical symptoms.^16^ However, in our study, the alignment rates for medical symptom groups still varied greatly ranging from 22% to 75%. Within the symptom groups with significantly higher alignment rates, both InfoKids+ and the nbTS had higher rates of urgent (<4 hours) triages (Supplemental Table 4). One reason for the better performance of medical symptom groups could be explained by the eRSA being more cautious regarding signs often witnessed with medical chief concerns (eg, bad general appearance) (Supplemental Figure 1). However, the tool performed poorly in triaging surgical symptom groups, coinciding with the relatively low rates of urgent (<4 hours) triages by the reference standard compared with InfoKids+ (Supplemental Table 4).

The 22 symptom groups that were adopted from the InfoKids application into the InfoKids+ eRSA covered for most of the symptoms reported, with less than 1% of participants describing a nonattributable symptom (Supplemental Table 3). The allocation into medical and surgical chief complaint, however, might have led to some confusion, because laypersons may struggle to differentiate their child’s symptoms into one of these categories.

Sociodemographic factors, particularly age, chronic diseases, and parental birthplace, considerably influenced triage alignment. Foreign-born parents achieved a lower alignment rate, although our tool was available in 3 languages (German, French and English). Nevertheless, language barriers might have led to miscommunication and thus a reduced quality of healthcare, or, in our case, acuity level alignment, as a systematic review found.^17^ Further, a Swiss study found that patients from other nationalities had a higher proportion of emergency department consultations although having fewer measures of severity.^18^ This suggests that the cultural background might influence the perception of symptoms and thus influence the acuity level of such tools. InfoKids+ triage aligned notably better in children with chronic diseases, suggesting that parents of these children might have better health literacy. In addition, InfoKids+ triage aligned better in younger children, which can be explained by the eRSA, just like the ATS, being more cautious regarding younger patients.^10^^,^^13^

### Strengths and Limitations

The study’s strengths lay in being the first robust validation study against a nbTS for a pediatric acuity eRSA to guide PED use. It offers in-depth insights into decision patterns, facilitating marked enhancements to the tool before considering routine implementation. Additionally, our study’s focus on demographic factors has unveiled their critical impact on the performance of eRSAs. High participant consent rates indicated strong acceptability of decision support tools.

However, our study faced some limitations. Notably it included only participants already present at the PED, thereby skewing our sample toward more concerned parents, resulting in a potential confirmation bias. Further limitations include a potential selection bias owing to its single-location setting and a tendency to include more cases with an ATS score of ≤3, as well as a considerably older population. This deviation from the baseline population is explained by the fact that the study team tried to include as many cases as possible into each symptom group to allow for a broader validation. However, some symptom groups included only a small sample size, limiting meaningful interpretation. Furthermore, a quarter of approached individuals declined participation, primarily owing to personal preference against participation (Figure 2). Despite similar core demographic characteristics with the participants, limited data under nonconsent prevented assessing potential sources of bias (Supplemental Table 2).

Data collection mainly took place during the COVID-19 pandemic in Switzerland, possibly altering the PED population compared with that before COVID. Moreover, the proportion of well-educated parents (ie, university or higher technical college degree) was higher than in the general Swiss population (49% vs 30%).^19^ The lack of a follow-up questionnaire in our study limited our ability to evaluate the tool’s effectiveness beyond providing acuity level guidance. Specifically, we were unable to determine the tool’s influence on reducing PED consultations. Additionally, we did not explore additional soft factors (eg, parental stress) on InfoKids+’ use, which are known to contribute to PED use.^20^ Currently, there is no consensus on what constitutes urgent, nonurgent, and no emergency, and we based our index and reference definitions on logical considerations related to disease severity and availability of services.^21^ However, changing cutoffs for both the index test and reference standard might markedly change the performance metrics of InfoKids+ (Figure 1; Supplemental Table 1).

### Comparison With Previous Research

Our findings align with previous studies on online triage tools. However, the comparison with previous literature is notably limited by the fact that we assessed the performance among patients, rather than theoretical clinical vignettes.^4^^,^^16^^,^^22^, ^23^, ^24^, ^25^, ^26^, ^27^, ^28^ The overreferral tendency observed with InfoKids+ aligns with the risk-averse nature of such tools reported in other studies.^4^^,^^22^ Previous studies also highlighted the importance of sociodemographic factors, such as age and education level, on the usage and performance of e-health tools.^29^

### Potential of Artificial Intelligence

Data-driven and continuous learning approaches such as machine learning and foundational models provide an opportunity to refine rule-based tools like InfoKids+ and incorporate performance feedback. For instance, a deep learning approach could consider more complex combinations of inputs to make finer-grained predictions better adapting to the differences uncovered by the subgroup analysis. Nonetheless, accuracy is vital in emergency triage, and, thus, it is essential to not only build the model on a trusted data source but also integrate it into a responsive framework that allows continuous adaptation and validation.^7^^,^^30^

## Conclusion

In summary, our study on the pediatric eRSA InfoKids+ uncovered notable discrepancies between its algorithmic triage and conventional nurse-based triage, alongside considerable variability in performance across different symptom categories. This finding is particularly striking given that InfoKids+ was designed on the basis of an established parental guidance application. Our results highlight the critical need for rigorous validation of such tools for accuracy and safety before public release, especially with the rise of artificial intelligence. We also observed a marked influence of sociodemographic factors on performance, emphasizing the importance of considering a user-centered design of such tools. Although InfoKids+ reported promise in facilitating patient pretriage during high-demand periods in PEDs, it is essential to conduct a thorough evaluation to ensure these tools do not inadvertently cause harm or resource misallocation.

## Potential Competing Interests

The authors report no competing interests.

## Ethics Statement

This study was approved by the Cantonal Research Ethics Committee of the Canton of Bern (project number 2019–02280) and was registered in the Registry of all Projects in Switzerland (RAPS). This study was conducted in accordance with the ethical standards of the Ethics Committee and with the principles of the Declaration of Helsinki.
