# Supplementary material for: InfoKids+: A Validation Study of a Pediatric Acuity Risk Stratification Algorithm
Source: Mayo Clin Proc Digit Health. 2025 Apr 15;3(2):100220. doi: 10.1016/j.mcpdig.2025.100220 (PMC12190946; doi:10.1016/j.mcpdig.2025.100220)
Supplement: Supplementary Material [file mmc1.docx]

**InfoKids+: A validation study of a paediatric acuity risk stratification algorithm**

**Supplementary Material**

**List of contents**

- Table S1. Supplemental 3x3 cross-tabulations Page 2
- Table S2. Comparison between the InfoKids+ study population and Participants declining informed

consent and the InfoKids+ study population and all PED patients from June 3, 2020 to January 31, 2022 Page 3

- Table S3. Attribution of symptom group “something else” Page 4
- Table S4. Percentage of “Urgent (<4h)” triage by the reference standard per chief complaint

and symptom group Page 5

- Figure S1. Simplified flowchart of the InfoKids+ eRSA Page 6

**Table S1. Supplemental 3x3 cross-tabulations**

| 3x3 cross tabulation without directly assigning worrying ^a^ as high acuity | | | | | | | | | X^2^ | 93.475 |
| --- | --- | --- | --- | --- | --- | --- | --- | --- | --- | --- |
| Reference standard  Index test | **Urgent (<4h)** | | **Non-urgent (<24h)** | | **No emergency (≥24h)** | | **Total** | | ***P*** | <.001 |
|  |  |  |  |  |  |  |  |  | **κ_lw_ ^b^ (95% CI)** | 0.086 (0.058 0.114) |
| Urgent (<4h) | 710 | (35.7%) | 622 | (31.3%) | 272 | (13.7%) | 1604 | (80.6%) |  |  |
| Non-urgent (<24h) | 22 | (1.1%) | 122 | (6.1%) | 15 | (0.8%) | 159 | (8.0%) |  |  |
| No emergency (≥24h) | 78 | (3.9%) | 99 | (5.0%) | 50 | (2.5%) | 227 | (11.4%) |  |  |
| Total | 810 | (40.7%) | 843 | (42.4%) | 337 | (16.9%) | 1990 | (100.0%) |  |  |

| 3x3 cross tabulation without directly assigning worrying ^a^ or high pain ^c^ as high acuity | | | | | | | | | X^2^ | 103.932 |
| --- | --- | --- | --- | --- | --- | --- | --- | --- | --- | --- |
| Reference standard  Index test | **Urgent (<4h)** | | **Non-urgent (<24h)** | | **No emergency (≥24h)** | | **Total** | | ***P*** | <.001 |
|  |  |  |  |  |  |  |  |  | **κ_lw_ ^b^ (95% CI)** | 0.120 (0.087 0.153) |
| Urgent (<4h) | 599 | (30.1%) | 520 | (26.1%) | 197 | (9.9%) | 1316 | (66.1%) |  |  |
| Non-urgent (<24h) | 38 | (1.9%) | 145 | (7.3%) | 22 | (1.1%) | 205 | (10.3%) |  |  |
| No emergency (≥24h) | 173 | (8.7%) | 178 | (8.9%) | 118 | (5.9%) | 469 | (23.6%) |  |  |
| Total | 810 | (40.7%) | 843 | (42.4%) | 337 | (16.9%) | 1990 | (100.0%) |  |  |

| 3x3 cross tabulation without directly assigning worrying ^a^ high pain ^c^ or worsening condition ^d^ as high acuity | | | | | | | | | X^2^ | 101.942 |
| --- | --- | --- | --- | --- | --- | --- | --- | --- | --- | --- |
| Reference standard  Index test | **Urgent (<4h)** | | **Non-urgent (<24h)** | | **No emergency (≥24h)** | | **Total** | | ***P*** | <.001 |
|  |  |  |  |  |  |  |  |  | **κ_lw_ ^b^ (95% CI)** | 0.114 (0.081 0.148) |
| Urgent (<4h) | 550 | (27.6%) | 476 | (23.9%) | 179 | (9.0%) | 1205 | (60.5%) |  |  |
| Non-urgent (<24h) | 51 | (2.6%) | 169 | (8.5%) | 28 | (1.4%) | 248 | (12.5%) |  |  |
| No emergency (≥24h) | 209 | (10.5%) | 198 | (10.0%) | 130 | (6.5%) | 537 | (27.0%) |  |  |
| Total | 810 | (40.7%) | 843 | (42.4%) | 337 | (16.9%) | 1990 | (100.0%) |  |  |

^a^ A rating exceeding 5 when answering the question “How worried are you about your child on a scale from 0 to 10?”

^b^ Linear weighted Kappa with the following levels of agreement: < 0 No agreement; 0.00 – 0.20 Slight agreement; 0.21 – 0.40 Fair agreement; 0.41 – 0.60 Moderate agreement; 0.61 – 0.80 Substantial agreement; > 0.81 almost perfect agreement

^c^ A rating of 9 or higher when answering the question “How do you rate your child’s pain on a scale from 0 to 10?”

^d^ Answering the question “Is your child’s condition getting worse?” with “yes”

**Table S2. Comparison between the InfoKids+ study population and Participants declining informed consent and the InfoKids+ study population and all PED patients from June 3, 2020 to January 31, 2022**

|  |  | InfoKids+ N=1990 N (%) | | No informed consent  N=633 (23 missing) N(%) | | InfoKids+ vs.  No informed consent | | All patients N=43483 N (%) | | InfoKids+ vs.  All patients | |
| --- | --- | --- | --- | --- | --- | --- | --- | --- | --- | --- | --- |
| Age | |  | |  | | **t-test** | ***P*** |  | | **t-test** | ***P*** |
|  | Mean | 79.48 | (±2.54 95%CI) | 77.32 | (±4.70 95%CI) | -0.9 | .35 | 67.14 | (±0.51 95%CI) | -10.4 | **<.001** |
|  | SD (min max) | 57.81 | (0 199) | 60.32 | (0 225) |  |  | 53.99 | (0 225) |  |  |
|  | Median (Q1 Q3) | 70 | (25 130) |  | (23 131) |  |  | 52 | (21 107) |  |  |
| Sex | |  |  |  |  | **X^2^** | ***P*** |  |  | **X^2^** | ***P*** |
|  | Male | 1067 | (53.6%) | 340 | (53.7%) | 0.0 | .97 | 23876 | (54.9%) | 1.3 | .26 |
|  | Female | 923 | (46.4%) | 293 | (46.3%) |  |  | 19607 | (45.1%) |  |  |
| ATS Score | |  |  |  |  | **X^2^** | ***P*** |  |  | **X^2^** | ***P*** |
|  | 1 | 16 | (0.8%) | 6 | (1.0%) | 8.1 | .15 | 343 | (0.8%) | 234.6 | **<.001** |
|  | 2 | 245 | (12.3%) | 86 | (13.6%) |  |  | 3794 | (8.7%) |  |  |
|  | 3 | 549 | (27.6%) | 159 | (25.1%) |  |  | 8661 | (19.6%) |  |  |
|  | 4 | 701 | (35.2%) | 207 | (32.7%) |  |  | 13504 | (31.1%) |  |  |
|  | 5 | 479 | (24.1%) | 174 | (27.5%) |  |  | 15897 | (36.6%) |  |  |
|  | FT | 0 | (0.0%) | 1 | (0.2%) |  |  | 1176 | (2.7%) |  |  |
|  | Missing | 0 | (0.0%) | 0 | (0.0%) |  |  | 108 | (0.3%) |  |  |

**Table S3. Attribution of symptom group “something else”**

| Chief complaint “something else”  Attribution | | Medical chief complaint N = 155 N (%) | | Surgical chief complaint N = 178 N (%) | |
| --- | --- | --- | --- | --- | --- |
| Medical chief complaint | | 150 | (96.8%) | 88 | (49.4%) |
|  | **Musculoskeletal complaints** | 23 | (14.8%) | 18 | (10.1%) |
|  | **Diabetes (Pees and drinks a lot)** | 4 | (2.6%) | 0 | (0.0%) |
|  | **ORL complaints (including ophthalmological complaints)** | 14 | (9.0%) | 12 | (6.7%) |
|  | **Gastrointestinal complaints** | 36 | (23.2%) | 4 | (2.3%) |
|  | **Psychiatric complaints** | 2 | (1.3%) | 1 | (0.6%) |
|  | **Fever** | 2 | (1.3%) | 1 | (0.6%) |
|  | **Headache** | 1 | (0.7%) | 12 | (6.7%) |
|  | **Malaise or convulsion (including loss of consciousness)** | 19 | (12.3%) | 18 | (10.1%) |
|  | **Dermatological complaints** | 14 | (9.0%) | 13 | (7.3%) |
|  | **Cough or difficulty breathing** | 4 | (2.6%) | 2 | (1.1%) |
|  | **Urogenital complaints** | 10 | (6.5%) | 1 | (0.6%) |
|  | **Something else** | 21 | (13.6%) | 4 | (2.3%) |
|  | **COVID-Test** | 0 | (0.0%) | 0 | (0.0%) |
| Surgical complaints | | 2 | (1.3%) | 80 | (44.9%) |
|  | **Ingestion** | 0 | (0.0%) | 1 | (0.6%) |
|  | **Burn injuries** | 0 | (0.0%) | 1 | (0.6%) |
|  | **Cut injuries** | 0 | (0.0%) | 17 | (9.6%) |
|  | **Injured tooth** | 0 | (0.0%) | 0 | (0.0%) |
|  | **Electric shock** | 0 | (0.0%) | 0 | (0.0%) |
|  | **Bite wounds** | 0 | (0.0%) | 0 | (0.0%) |
|  | **Sting** | 0 | (0.0%) | 2 | (1.1%) |
|  | **Fracture or injury** | 2 | (1.3%) | 57 | (32.0%) |
|  | **Something else** | 0 | (0.0%) | 2 | (1.1%) |
| Non attributable | | 3 | (1.9%) |  | 10 |

**Table S4. Percentage of “Urgent (<4h)” triage by the reference standard per chief complaint and symptom group**

|  | | | Total  N = 1990 N (%) | | Triaged “Urgent” by reference standard  N = 810 N (%) | | Triaged “Non-urgent” or “No emergency” by reference standard  N = 1180 N (%) | | X^2^ | *P* |
| --- | --- | --- | --- | --- | --- | --- | --- | --- | --- | --- |
| Chief complaints | | |  |  |  |  |  |  |  |  |
|  | **Medical chief complaint ^a^** | | 1108 | (55.7%) | 556 | (50.2%) | 552 | (49.8%) | 93.026 | **<.001 ^b^** |
|  |  | **Musculoskeletal complaints** | 60 | (3.0%) | 15 | (25.0%) | 45 | (75.0%) | 6.321 | **.01 ^c^** |
|  |  | **Diabetes (Pees and drinks a lot)** | 13 | (0.7%) | 6 | (46.2%) | 7 | (53.8%) | 0.161 | .69 |
|  |  | **ORL complaints (including ophthalmological complaints)** | 156 | (7.8%) | 60 | (38.5%) | 96 | (61.5%) | 0.353 | .55 |
|  |  | **Gastrointestinal complaints** | 374 | (18.8%) | 167 | (44.7%) | 207 | (55.3%) | 2.976 | .09 |
|  |  | **Psychiatric complaints** | 16 | (0.8%) | 12 | (75.0%) | 4 | (25.0%) | 7.861 | **.005 ^b^** |
|  |  | **Fever** | 359 | (18.0%) | 179 | (49.9%) | 180 | (50.1%) | 15.218 | **.001 ^b^** |
|  |  | **Headache** | 131 | (6.6%) | 58 | (44.3%) | 73 | (55.7%) | 0.741 | .39 |
|  |  | **Malaise or convulsion (including loss of consciousness)** | 126 | (6.3%) | 91 | (72.2%) | 35 | (27.8%) | 55.367 | **<.001 ^b^** |
|  |  | **Dermatological complaints** | 80 | (4.0%) | 38 | (47.5%) | 42 | (52.5%) | 1.595 | .21 |
|  |  | **Cough or difficulty breathing** | 225 | (11.3%) | 142 | (63.1%) | 83 | (36.9%) | 52.774 | **<.001 ^b^** |
|  |  | **Urogenital complaints** | 70 | (3.5%) | 35 | (50.0%) | 35 | (50.0%) | 2.598 | .11 |
|  |  | **Something else** | 155 | (7.8%) | 87 | (56.1%) | 68 | (43.9%) | 16.572 | **<.001 ^b^** |
|  |  | **COVID-Test** | 27 | (1.4%) | 11 | (40.7%) | 16 | (59.3%) | 0.000 | .99 |
|  | **Surgical complaints ^a^** | | 882 | (44.3%) | 254 | (28.8%) | 628 | (71.2%) | 93.026 | **<.001 ^c^** |
|  |  | **Ingestion** | 31 | (1.6%) | 25 | (80.6%) | 6 | (19.4%) | 20.815 | **<.001 ^b^** |
|  |  | **Burn injuries** | 13 | (0.7%) | 3 | (23.1%) | 10 | (76.9%) | 1.685 | .19 |
|  |  | **Cut injuries** | 63 | (3.2%) | 12 | (19.1%) | 51 | (80.9%) | 12.642 | **<.001 ^c^** |
|  |  | **Injured tooth** | 20 | (1.0%) | 8 | (40.0%) | 12 | (60.0%) | 0.004 | .95 |
|  |  | **Electric shock** | 2 | (0.1%) | 1 | (50.0%) | 1 | (50.0%) | 0.072 | .79 |
|  |  | **Bite wounds** | 10 | (0.5%) | 2 | (20.0%) | 8 | (80.0%) | 1.785 | .18 |
|  |  | **Sting** | 7 | (0.4%) | 1 | (14.3%) | 6 | (85.7%) | 2.031 | .15 |
|  |  | **Fracture or injury** | 625 | (31.4%) | 153 | (24.5%) | 472 | (75.5%) | 99.364 | **<.001 ^c^** |
|  |  | **Something else** | 178 | (8.9%) | 73 | (41.0%) | 105 | (59.0%) | 0.008 | .93 |

^a^ Although the question between medical and surgical chief complaint was a single-choice question, the individual symptom groups per chief complaint (medical or surgical) were part of a multiple choice question.

^b^ Statistical significant more “Urgent (<4h)” triages by the reference standard compared to all other cases

^c^ Statistical significant less “Urgent (<4h)” triages by the reference standard compared to all other cases


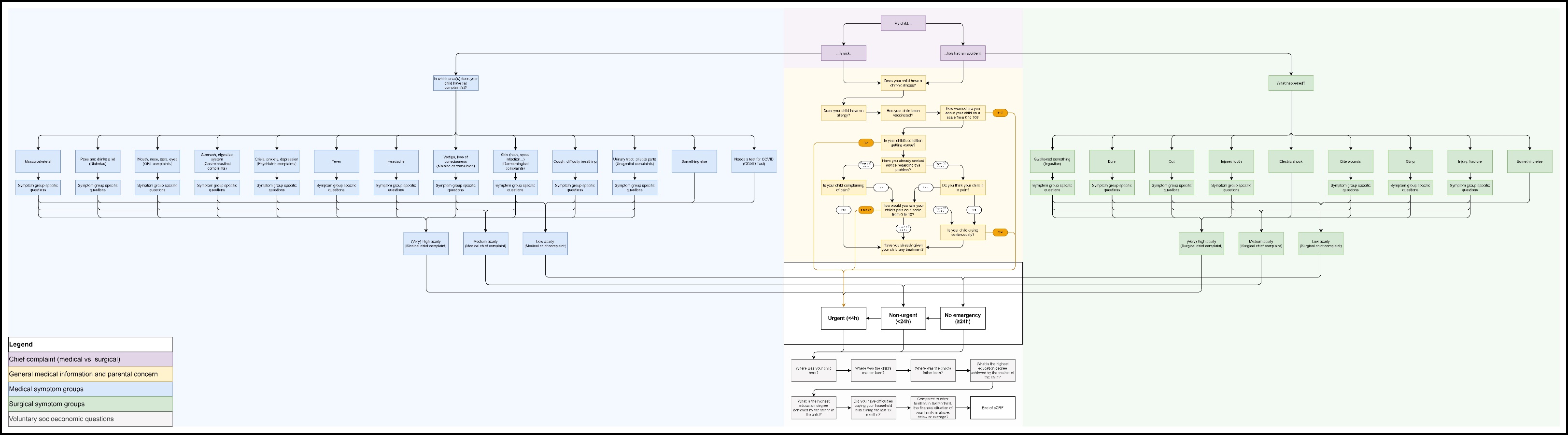


**Figure S1. Simplified flowchart of the InfoKids+ eRSA**
